# The diagnostic value of D-dimer in acute aortic dissection: a meta-analysis

**DOI:** 10.1186/s13019-021-01726-1

**Published:** 2021-11-27

**Authors:** Jian Yao, Tao Bai, Bo Yang, Lizhong Sun

**Affiliations:** Department of Cardiovascular Surgery, Beijing Anzhen Hospital of Capital Medical University and Beijing Institute of Heart, Lung and Blood Vessel Diseases, No. 2 Anzhen Road, Chaoyang District, Beijing, 100029 China

**Keywords:** D-dimer, Acute aortic dissection, Meta-analysis, Diagnostic value

## Abstract

**Objective:**

This study aims to evaluate the diagnostic value of D-dimer for acute aortic dissection (AAD) by the method of meta-analysis.

**Methods:**

PubMed, Cochrane Library, Web of Science, Embase, China National Knowledge Infrastructure (CNKI), and Wanfang databases from the establishment of the databases to December 2020 were systematically searched, and the Quality Assessment of Diagnostic Accuracy Studies-2 (QUADAS-2) system was used to evaluate the quality of the literature. STATA 15.0 software was applied to calculate the pooled sensitivity, specificity, diagnostic odds ratio (DOR), positive likelihood ratio (+LR), negative likelihood ratio (−LR) to draw summary receiver operating characteristics (SROC) curve and calculate the area under the curve (AUC). Meta-regression and subgroup analyses were used to explore the source of heterogeneity.

**Results:**

A total of 16 clinical studies were enrolled in this study, including 1135 patients. The results of the meta-analysis showed that the pooled sensitivity was 0.96 (95% CI 0.91–0.98), the pooled specificity was 0.70 (95% CI 0.57–0.81), and the pooled DOR was 56.57 (95% CI 25.11–127.44), the pooled +LR was 3.25 (95% CI 2.18–4.85), the pooled −LR was 0.06 (95% CI 0.03–0.12), and the AUC was 0.94 (95% CI 0.91–0.95). Meta-regression and subgroup analysis results showed that publication year, sample size and cutoff value might be sources of heterogeneity. When the concentration of D-dimer was less than or equal to 500 ng/ml, the sensitivity significantly increased.

**Conclusion:**

D-dimer has an excellent diagnostic value for AAD. It is a useful tool for detecting suspected AAD because of the excellent pooled sensitivity. D-dimer ≤ 500 ng/ml increases the potential to identify the suspected patients with AAD.

## Introduction

Acute aortic dissection (AAD) is a dangerous cardiovascular disease in which blood passes through the aortic intimal tear and enters the aortic wall to separate it, forming a true and false cavity [1]. Epidemiological results show that the annual incidence of AAD is six people per 100,000 people [2]with a high risk of rupture in AAD and high potential mortality rate [3]. Research reports have shown that the mortality rate within 24 h of the onset of AAD is 35%, and the mortality rate is 50% within 48 h [4]. Therefore, the early diagnosis and treatment of AAD are essential for patient survival.

At present, computerized tomography (CT), transthoracic and transesophageal echocardiography, magnetic resonance angiography (MRA), and digital subtraction angiography (DSA) are mainly used to help diagnose AAD [5, 6]. However, these diagnostic techniques are usually time-consuming, limited in some hospitals that lack large diagnostic equipment, which are prone to misdiagnosis and missed diagnoses [7]. As AAD symptoms are not specific, it is easy to be confused with other chest pain-based diseases such as acute myocardial infarction. It is impossible for doctors to perform enhanced CT examinations on all suspected patients at the time of diagnosis. Therefore, it is urgent to explore a convenient, fast, effective and safe diagnosis method for AAD.

Biomarkers are easy to analyze through blood tests and become a substitute tool for clinical disease diagnosis. It is reported that elevated levels of fibrinogen/fibrin degradation products, soluble elastin fragments (sELAF), tenascin-C [8–10], smooth muscle myosin heavy chain (sm-MHC) [11] are found when AAD occurs. However, the detection system and reference range of these potential biomarkers are not completely effective in diagnosing AAD. D-dimer is a specific degradation product produced by plasmin hydrolysis, and the level of D-dimer can be detected through peripheral blood [12]. In 2014, the European Society of Cardiology (ESC) included D-dimer in the guidelines for the diagnosis and treatment of aortic diseases, and pointed out that if the test result of D-dimer is negative, patients with low levels of possible AAD can be ruled out, and imaging examinations are not required [13]. Many studies have confirmed that D-dimer levels increase in AAD [14, 15], which is expected to be a biomarker to assist in the diagnosis of AAD. A meta-analysis by Asha et al. [15] in 2015 validated a good diagnostic value of D-dimer for acute aortic coarctation. However, there are still more studies published since 2015 [16–18].

The aim of the present meta-analysis is to explore the diagnostic value of D dimer in acute aortic dissection. Statistical data such as sensitivity, specificity, positive likelihood ratio (+LR), negative likelihood ratio (−LR), diagnostic odds ratio (DOR), summary receiver operating characteristic (SROC) curve and area under the curve (AUC) were collected. The related heterogeneity, publication bias and sensitivity were evaluated to clarify the accuracy of D-dimer's diagnosis for AAD, and reduce misdiagnosis and missed diagnosis of AAD.

## Methods

### Retrieval strategy

According to the designed flow diagram, literature retrieval was performed in English databases PubMed, Cochrane Library, Web of Science, and Embase as well as Chinese databases China National Knowledge Infrastructure (CNKI) and Wanfang databases. The search date starts from inception to December 2020, and the search language is not limited. Keywords "aortic dissection", "D-dimer" and "diagnosis" were used to evaluate the diagnostic value of D-dimer for AAD. Two researchers conducted literature searches independently.

### Literature selection

#### Inclusion criteria

(1) AAD was diagnosed; (2) D-dimer level was measured; (3) Human study; (4) The results of true positives, true negatives, false positives, and false negatives were reported or can be calculated.

#### Exclusion criteria

(1) Articles of review, case report, animal experiment research and comment type; (2) Repeated publications (only the research with the most complete data was selected); (3) Articles with only abstract or with insufficient important information such as *P* value, 95% confidence interval information or the diagnostic sensitivity and specificity cannot be extracted.

### Literature evaluation

The quality of the included literature was evaluated by two investigators using the Quality Assessment of Diagnostic Accuracy Studies-2 (QUADAS-2) risk assessment tool [19]. When the results were inconsistent, the decision was made through consultation or discussion with the third investigator. The four components of the QUADAS-2 (case selection, test to be evaluated, gold standard, case flow and progress) are subject to the risk of bias evaluation.

### Data extraction

The extracted data from the published studies include: (1) First author, publication year, number of cases, age, gender, D-dimer concentration, and cutoff value; (2) True positive, false positive, false negative, and true negative.

### Statistical analysis

The literature data management software Stata 15.0 (Stata Corporation, USA) was used to analyze the data of the included literature. The I^2^ test was used to test the heterogeneity across studies. If I^2^ < 50%, it was considered that there was no heterogeneity; otherwise, it was considered that there was heterogeneity. The threshold effect was judged based on the typical "shoulder-arm" shape in the SROC curve and the correlation between the logarithm of sensitivity and the logarithm of 1-specificity. The bivariate mixed-effects model was used to pool sensitivity, specificity, DOR,  +LR, and −LR. The AUC value of the ROC curve was calculated. The closer the AUC value is to 1, the higher the diagnostic power is considered. The publication bias is shown using Deeks’ funnel plot. If *P* < 0.05, it was considered that there was publication bias. Meta-regression and subgroup analysis was used to explore the sources of heterogeneity. Finally, a sensitivity analysis was used to assess the robustness of the findings.

## Results

### Literature search results and basic information of included studies

**Fig. 1:**
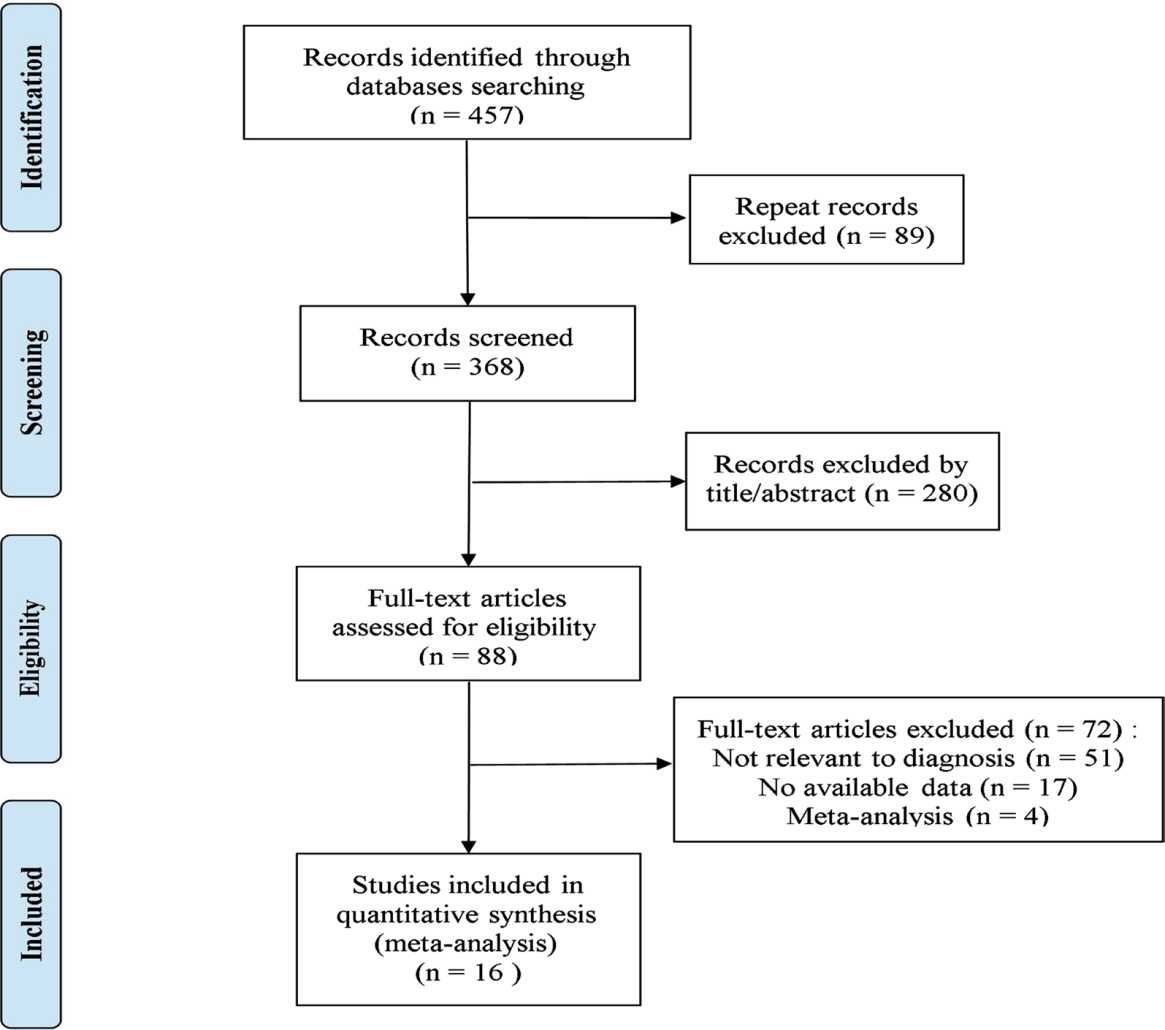
A flow chart of the study selection process

The two researchers searched the relevant databases according to the pre-established literature search strategy, and obtained 457 related documents, including 89 duplicate documents. A total of 280 irrelevant articles were excluded by reading the title and abstract. After reading the full text to check the completeness and correctness of the data, 72 articles were excluded, and 16 studies [16–18, 20–32] that met the inclusion criteria were included in this meta-analysis. Figure 1 shows the specific literature screening flow diagram, and the basic data of included studies and related data of diagnostic tests are displayed in Table.1.

**Table 1:** Characteristics of included studies

First author	Year	Country	Case (control)	Detection method	Cut-off value	TP	FP	FN	TN	Sensitivity (%)	Specificity (%)
Eggebrecht [20]	2004	Germany	16 (80)	Latex-enhanced turbidimetric test	0.626 µg/mL	16	0	58	22	100	73
Akutsu [21]	2005	Japan	30 (48)	Latex agglutination test	0.5 µg/mL	30	0	26	22	100	54
Hazui [22]	2005	Japan	29 (49)	Latex agglutination test	0.8 or 0.9 μg/ml	27	2	45	4	93.1	91.8
Ohlmann [23]	2006	France	94 (94)	Immunoturbidimetric assay	400 ng/mL	93	1	32	62	99	34
Sbarouni [24]	2007	Greece	18 (29)	ELISA	700 ng/ml	17	1	17	12	94	59
Xue [25]	2007	China	16 (27)	Immunoturbidimetric assay	0.4 µg/mL	16	0	18	9	100	66
Suzuki [26]	2009	Japan	87 (133)	Triage D-dimer test	500 ng/mL	84	3	62	71	96.6	46.6
Fan QK [27]	2010	China	107 (153)	Immunoturbidimetric assay	0.17 µg/mL	106	1	47	106	99.2	30.9
Sakamoto [28]	2011	Japan	35 (228)	Latex-enhanced turbidimetric test	5 µg/mL	24	11	206	22	68.4	90.3
Nazerian [29]	2014	Italy	233 (802)	Latex agglutination test	500 ng/ml	229	4	288	514	98.3	35.9
Peng [30]	2015	China	35 (51)	ELISA	2.11 µg/mL	28	7	46	5	80	90.21
Yoshimuta [31]	2015	Japan	9 (1227)	Latex agglutination test	6.9 μg/ml	9	0	1163	64	100	94.8
Gorla [32]	2015	Germany	159 (72)	Microparticle-enhanced immunoassay	0.5 mg/L	157	2	48	24	99	67
Sakamoto [16]	2016	Japan	5 (235)	NR	5.0 μg/ml	5	0	215	20	100	91.7
Xiao [17]	2016	China	60 (60)	Automatic coagulation instrument	1.435 mg/L	56	4	41	19	93.33	68.33
Dong [18]	2017	China	202 (150)	Immune nephelometry	NR	139	63	91	59	68.8	60.9

*ELISA* enzyme linked immunosorbent assay, *TP* true positive, *FP* false positive, *FN* false negative, *TN* true negative, *NR* not reported

### QUADAS-2 scores

**Fig. 2:**
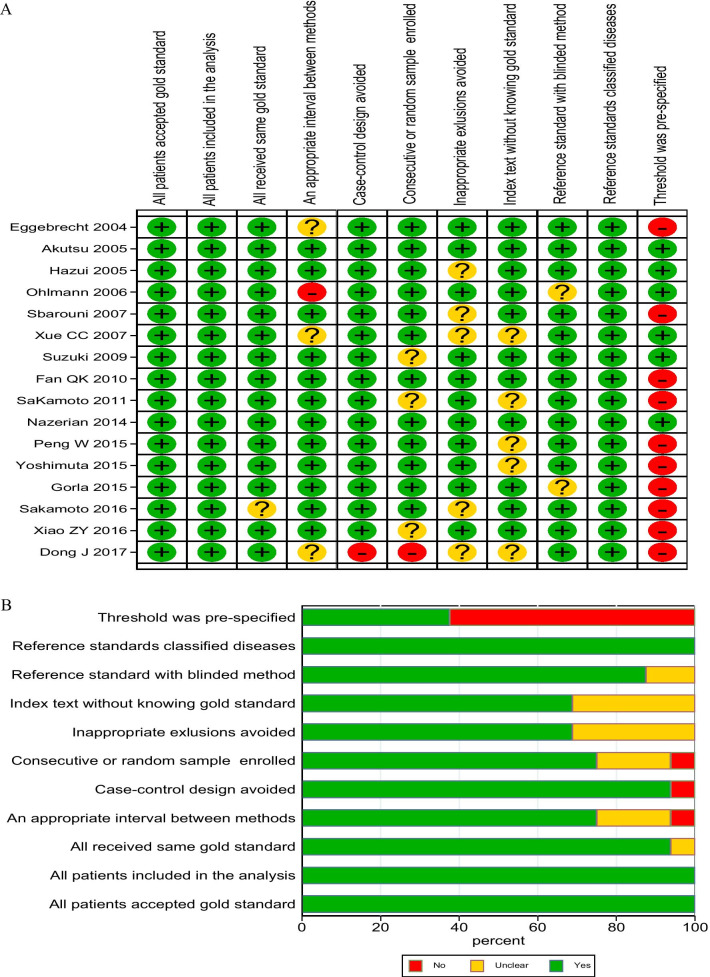
Results of literature quality evaluation. **a** Results of the evaluation of each study according to QUADAS-2. **b** Bar chart of quality score of diagnostic test literature. *QUADAS-2* Quality Assessment of Diagnostic Accuracy Studies-2

In the 16 selected studies, there were 1135 cases. After reading the literature carefully with the baseline characteristics of the included literature mastered, the questions in each part of the QUADAS-2 tool was answered using "yes", "no", or "unclear", the corresponding risk of bias was judged as "low", "high" and "uncertain". The results show that (Fig. 2a, b) the clinical studies included are of high quality. All cases were drawn continuously or randomly. The gold standard can correctly identify the target condition. However, the diagnostic cut-off values of some clinical studies were preset, and more than 50% were calculated through the SROC curve. Therefore, more than 50% of clinical studies had an uncertain bias in the interpretation of D-dimer test results.

### Meta-analysis results

**Fig. 3:**
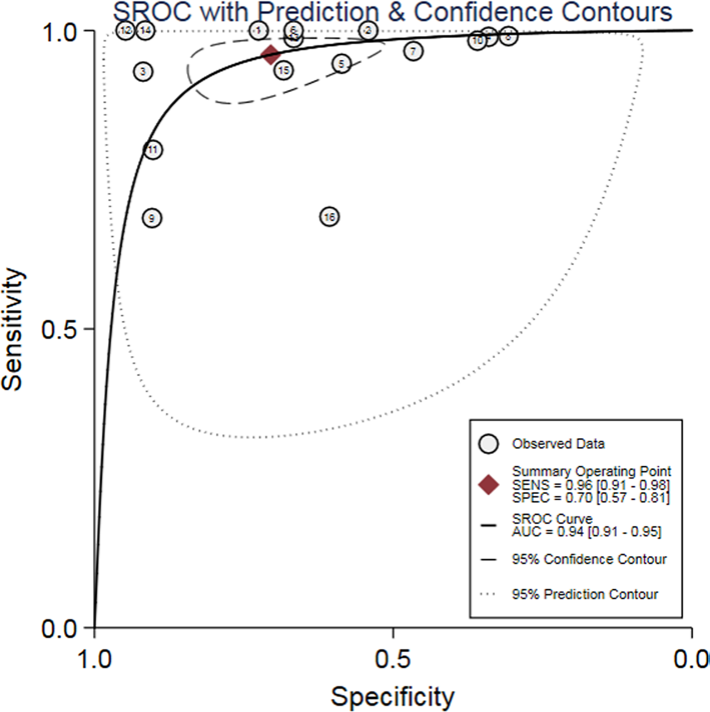
SROC curve for the accuracy of D-dimer in the diagnosis of acute aortic dissection. SROC curve: summary receiver operating characteristic curve; AUC: area under the curve

The SROC curve did not show the typical "shoulder-arm" shape (Fig. 3), while the Spearman correlation coefficient between the log of sensitivity and the log of 1-specificity was 0.0702(*P* = 0.796), which indicated that there was no threshold effect in this meta-analysis. As the heterogeneity across studies did not come from threshold effect, the following combination analysis could be performed.

**Fig. 4:**
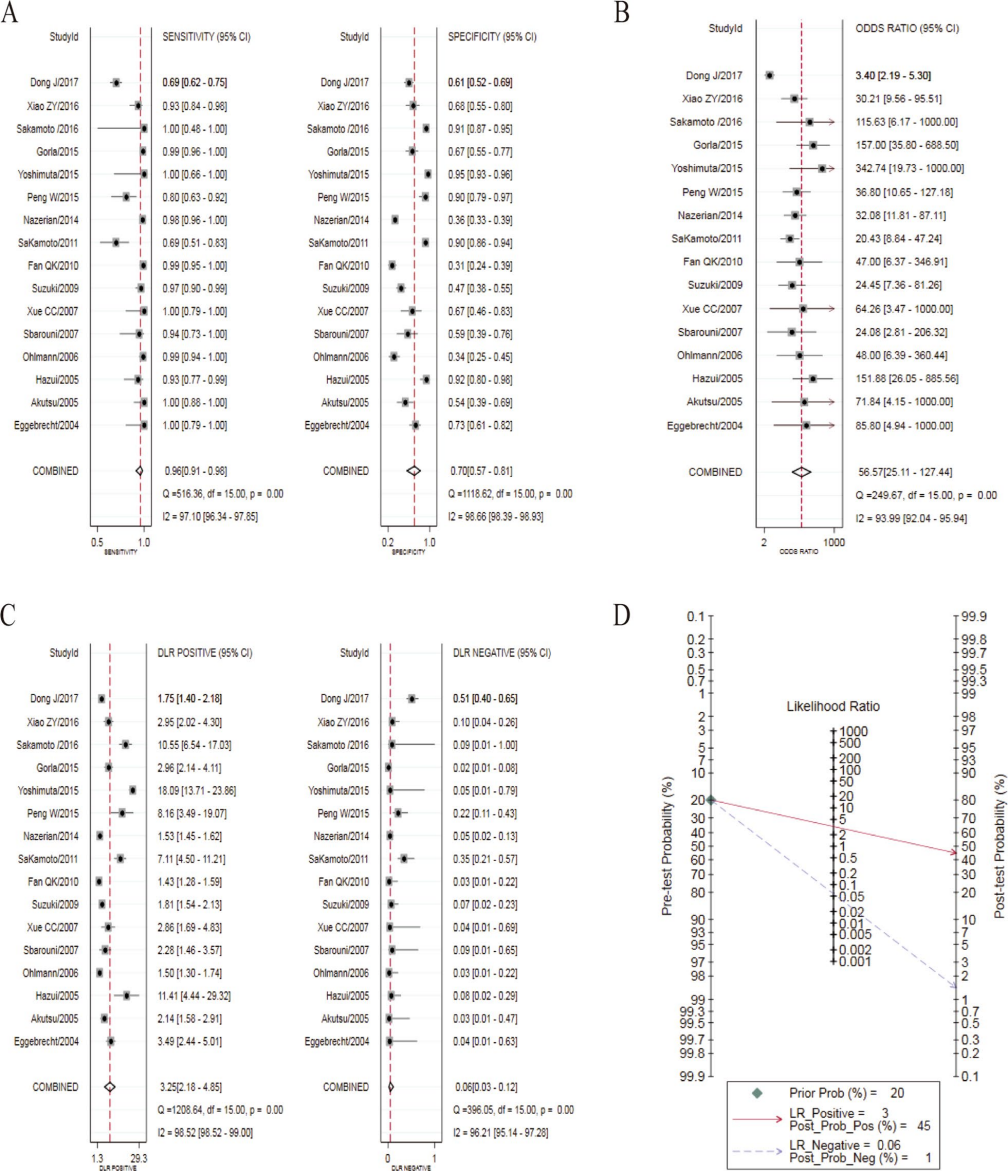
Forest plot of D-dimer for the diagnosis of acute aortic dissection. **a** sensitivity and specificity. **b** DOR. **c** +LR and −LR. **d** Fagan’s Nomogram. DOR, diagnostic odds ratio;  +LR, positive likelihood ratio; − LR, negative likelihood ratio

Heterogeneity analysis showed that there was heterogeneity in the pooled analysis of sensitivity (I^2^ = 97.10%, *P* < 0.01,) (Fig. 4a), specificity (I^2^ = 98.66%, *P* < 0.01) (Fig. 4a), DOR (I^2^ = 93.99%, *P* < 0.01) (Fig. 4b),  +LR (I^2^ = 98.52%, *P* < 0.01) (Fig. 4c), and −LR (I^2^ = 96.21%, *P* < 0.01) (Fig. 4c).

The summary analysis results of the bivariate mixed-effects model showed that, the diagnostic accuracy of D-dimer detection of AAD was as follows: the pooled sensitivity = 0.96 (95% CI 0.91–0.98) (Fig. 4a), the pooled specificity = 0.70 (95% CI 0.57–0.81) (Fig. 4a), the pooled DOR = 56.57 (95% CI 25.11–127.44) (Fig. 4b), the pooled +LR = 3.25 (95% CI 2.18–4.85) (Fig. 4c), the pooled −LR = 0.06 (95% CI 0.03–0.12) (Fig. 4c), and the AUC of the SROC curve was 0.94 (95% CI 0.91–0.95). The results of Fagan’s Nomogram showed that if the pre-test probability ratio was 20%, the post-test probability of +LR was 45%, while the post-test probability of −LR was 1% (Fig. 4d).

### Publication bias

**Fig. 5:**
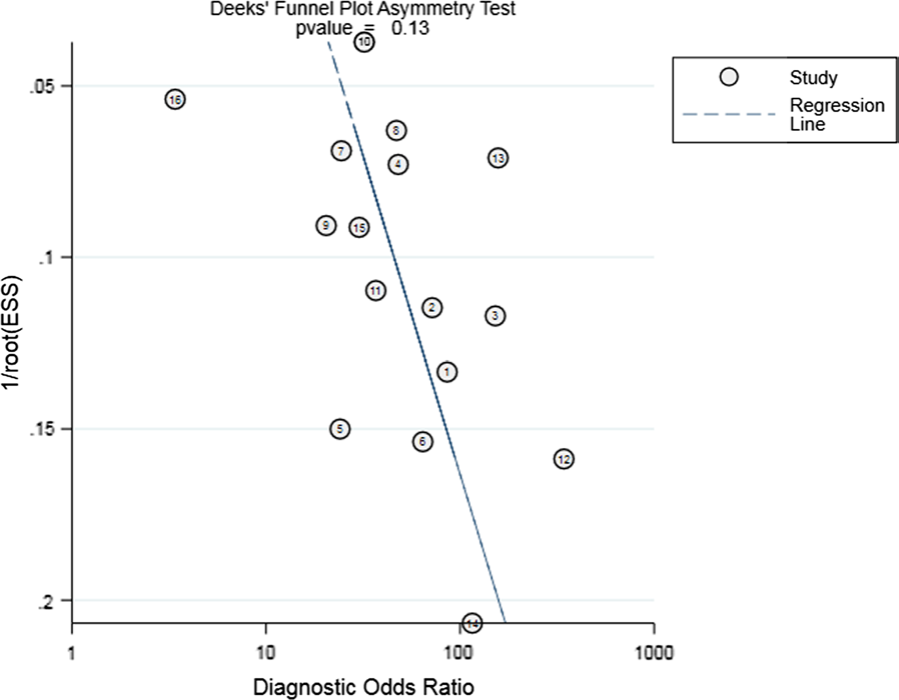
Funnel plot of D-dimer for the diagnosis of acute aortic dissection

The linear regression method was used to test the asymmetry of the funnel plot. The Deeks’ funnel plot was symmetrical, *P* value being 0.13, which indicated no publication bias (Fig. 5).

### Meta-regression and subgroup analysis

**Fig. 6:**
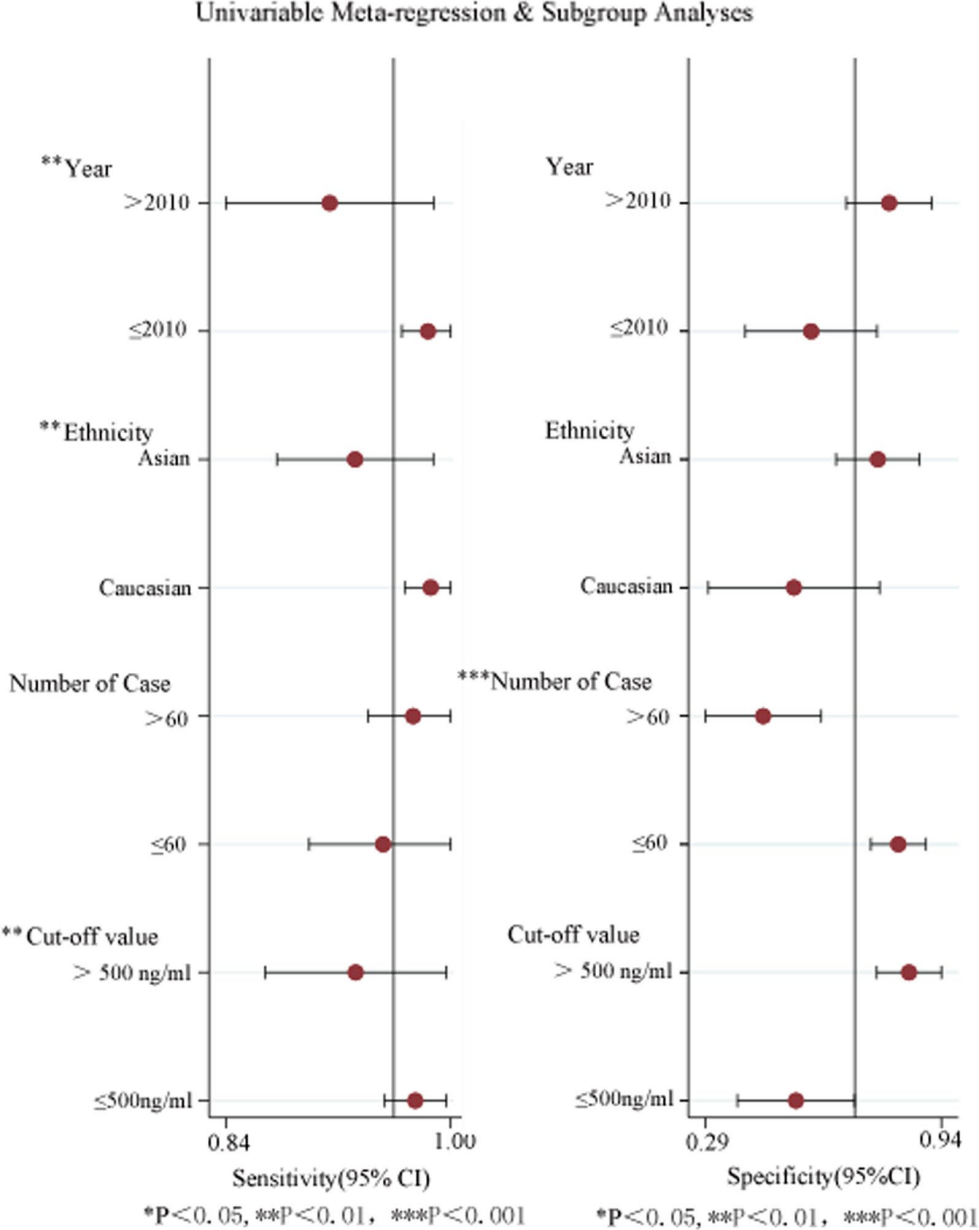
The results of meta-regression and subgroup analyses

To explore the sources of heterogeneity among the included studies, we conducted meta-regression and subgroup analysis. Article publication year, research area, sample size, and cut-off value were included in the analysis (Fig. 6). The results showed that the publication year, sample size, and cut-off value of the article led to differences in diagnostic value (*P* < 0.05), which may be the source of heterogeneity.

The subgroup analysis results showed that diagnostic criteria, such as the publication year ≤ 2010, Caucasian, and cut-off value ≤ 500 ng/mL have high sensitivity. The number of cases ≤ 60 has high specificity. These results showed that D-dimer had an excellent diagnostic performance for AAD. Because of its high sensitivity, it is an extremely useful tool for detecting suspected patients with AAD. Patients with AAD are more likely to be identified when the cut-off value was less than or equal to 500 ng/ml, while when the cut-off value was more than 500 ng/ml, it was more beneficial to exclude non-AAD populations.

### Sensitivity analysis

**Fig. 7:**
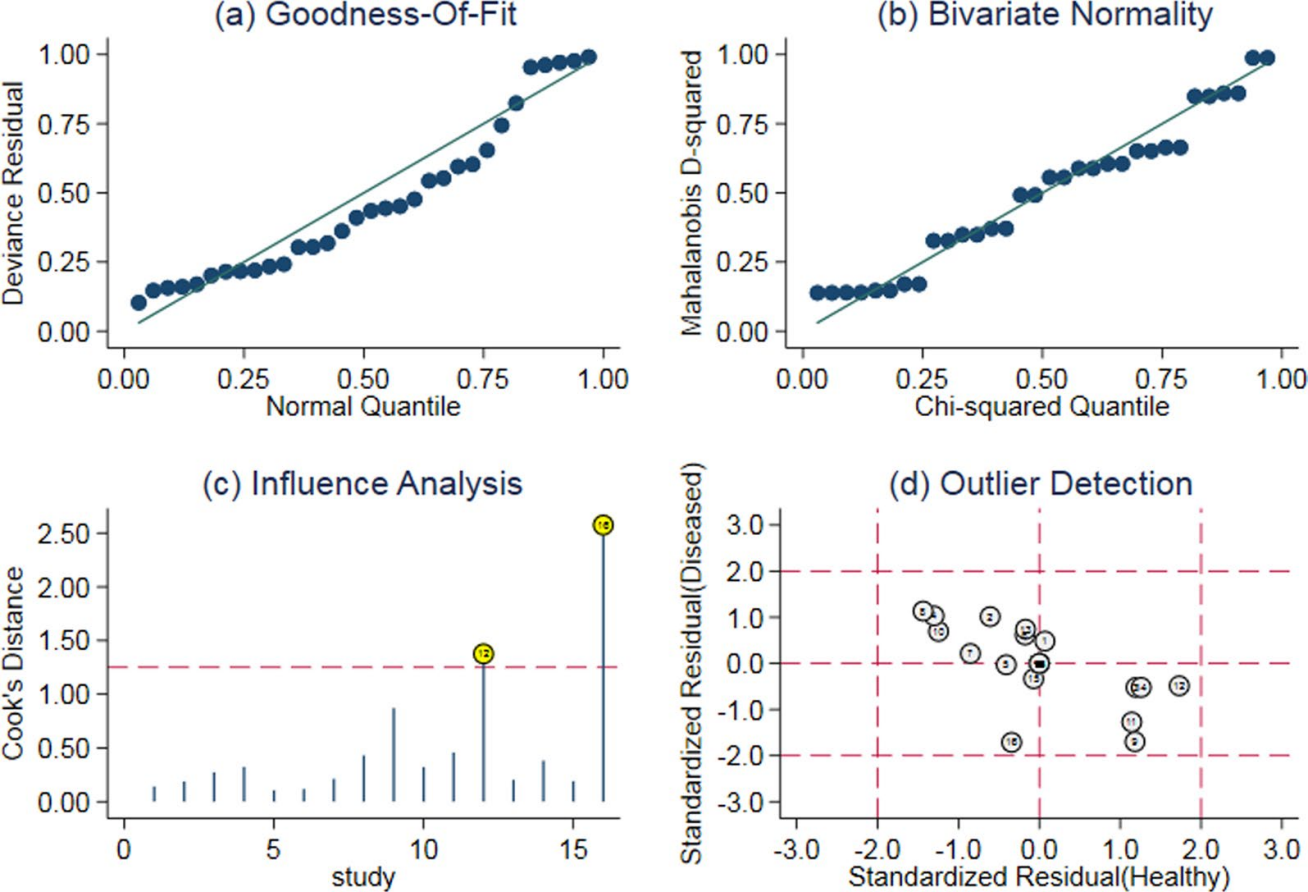
The results of sensitivity analysis

The sensitivity analysis results of D-dimer's diagnostic value showed a high degree of goodness of fit and normal bivariate (Fig. 7a, b). Sensitivity analysis found that two studies [18, 31] have larger statistical weights (Fig. 7c). After the outlier detection, these two studies did not show outliers (Fig. 7d). Further testing was conducted, after removing the two studies, the pooled sensitivity remained unchanged, the pooled specificity decreased from 0.70 to 0.68, the pooled DOR decreased from 56.57 to 49, the pooled +LR decreased from 3.25 to 3.0, the pooled −LR remained unchanged, and the AUC of the SROC curve decreased from 0.94 to 0.93. These data indicated that the pooled effect size of the reanalysis was relatively robust compared with the pooled results before the exclusion. The findings of this meta-analysis were robust.

## Discussion

The survival rate of AAD patients depends on their early diagnosis and timely and effective treatment. Evidences demonstrate that its early differential diagnosis is difficult [33, 34]. In recent years, studies have found that there exists a certain correlation between AAD and D-dimer, CRP, sm-MHC, sELAF [35–37]. Specifically, CRP is a prognostic biomarker of AAD [38]. The sm-MHC is not conducive to the diagnosis of AAD due to narrowed time window and complicated detection process [12]. The increase in sELAF level is related to the increase of age, and the detection time is longer [9]. Some studies have shown that D-dimer is a biomarker with high sensitivity and low specificity, which can be used as a tool to identify suspected AAD [39, 40].

This study conducted a systematic review of the included 16 clinical studies. The results showed that the pooled sensitivity and specificity of D-dimer in the diagnosis of AAD were 0.96 and 0.70, respectively. This suggests that the pooled sensitivity was excellent, the pooled specificity being moderate. The sensitivity and specificity are strongly influenced by the cut-off value. In the studies of Akutsu et al. [21] and Suzuki et al. [26], the cut-off values were set at 500 ng/ml, with sensitivities above 90% and specificities below 60%. In the studies of SaKamoto et al. [16] and Peng et al. [30], the cut-off values were 5 µg/mL and 2.11 µg/mL, respectively, far above 500 ng/ml, with sensitivities below 90% and specificities above 90%. In order to detect as many suspected patients with AAD as possible, the appropriate threshold is crucial. The likelihood ratio is a comprehensive index calculated by pooling sensitivity and specificity. According to reports,  +LR greater than 10 and −LR less than 0.1 have convincing accuracy [41]. The results of this study showed that the pooled +LR was 3.25 and the pooled −LR was 0.06, indicating that the probability of a positive diagnostic test being correctly judged was 3.25 times the probability of a false positive judgment, and the probability of a wrong judgment of negative being 0.06 times the probability of a correct judgment of negative. The DOR value ranges from 0 to infinity, and the higher the value, the stronger the diagnostic ability [42]. The results of this study showed that the pooled DOR was 56.57, suggesting that D-dimer is a biomarker for the diagnosis of AAD. Each point on the ROC curve represents the sensitivity and specificity of the corresponding critical value, which comprehensively reflects the diagnostic value of the diagnostic test for the target disease. The AUC value of the area between 0.93 and 0.96 is considered to have accurate diagnostic ability [43]. The AUC of this study was 0.94, which was close to 1, indicating that D-dimer has a high clinical value in the diagnosis of AAD. The publication bias and sensitivity analysis results of this study showed that the results of the study were reliable.

Meta-regression analysis and subgroup analysis showed that the publication year, ethnicity, sample size and cut-off value of the article have a significant impact on heterogeneity. The reason might be the inconsistent detection rate of positive results under different sample sizes and cut-off values. The difference of heterogeneity across publication years could be due to other reasons, such as differences in testing methods, population groups, etc. D-dimer was more sensitive to AAD detection in Caucasian population than Asian population, which might be caused by ethnic differences. The cut-off value was an significant source of heterogeneity. From the findings, the sensitivity was excellent and the specificity was not low when the D-dimer concentration was less than 500 ng/ml. This facilitates the identification of suspected patients with AAD.

In the meta-analysis published by Asha et al. [15] in 2015, 4 clinical studies with a cut-off value of 0.50 µg/mL containing 457 AAD patients were included for analysis. His results showed that the pooled sensitivity was 0.98, the pooled specificity was 0.419, the pooled +LR was 2.11, pooled −LR was 0.05 and concluded that a negative D-dimer result might help rule out AAD in low-risk patients. Our study included 16 clinical studies with a total of 1135 AAD patients. Compared with previous studies, the number of studies and sample size had increased, and the statistical results were more convincing. In addition, the research level of the included literature was relatively high, and the literature had been blindly extracted to minimize the inclusion bias. At the same time, the statistical indicators were tested for heterogeneity, which improved the internal authenticity of the research. Moreover, meta-regression and subgroup analysis were performed to discover and screen the main factors of heterogeneity, guiding significance for future research work.

The findings of this meta-analysis have significant clinical implications. The results of this study suggest that D-dimer can be used to differentiate AAD from other diseases with high sensitivity, which indicates high negative prediction in patients with AAD. D-dimer levels in patients with AAD are significantly higher than in other diseases in which chest pain is a prominent symptom (such as acute coronary syndrome), except acute pulmonary embolism [44, 45]. However, there are no accepted cut-off values to distinguish AAD from acute coronary syndrome in current studies [45, 46]. The similarity of AAD to acute coronary syndrome is a particular problem. Despite improvements in diagnostic measures, including imaging and biomarkers, misdiagnosis of AAD remains common [46]. It remains a critical topic to explore more appropriate biomarkers for the early diagnosis of AAD in current research. Moreover, D-dimer levels may have a strong correlation with the severity of the patients with AAD [47, 48]. Therefore, the cut-off values of D-dimer may be crucial for the differential diagnosis of AAD, as well as the severity of the disease. The present study provides a little hint in this regard. More researches are still needed on the cut-off values of D-dimer in the future. Unavoidably, there were some limitations in our study. (1) The diagnostic cut-off value of some clinical researches was preset, the diagnostic cut-off value was not calculated by SROC, so the preset diagnostic cut-off value was not the best diagnostic cut-off value for this clinical research. There may be a certain degree of bias in the judgment of positive results. (2) The clinical research in this project used a variety of methods to detect the concentration of D-dimer. Because different detection methods have different detection ranges and time, sensitivity and specificity, there may be a certain bias in the interpretation of positive results. (3) The research came from different countries, the demographic characteristics of the research objects in different regions were different, and the scientific research conditions and levels were also inconsistent, which affected the accuracy of diagnosis. Therefore, a large sample, random, blinded research design was used to study the correlation between D-dimer and AAD diagnosis and health economic evaluation was also needed to make the results more clinically meaningful.

In conclusion, this study revealed the relationship between D-dimer and AAD patients from the perspective of meta-analysis. The results showed that D-dimer had an excellent diagnostic performance in the diagnosis of AAD. D-dimer is a useful indicator for detecting suspected AAD because of the excellent pooled sensitivity. D-dimer ≤ 500 ng/ml increases the potential to identify the suspected patients with AAD. The findings of this study have yet to be confirmed by a more extensive and rigorously designed multi-center study.

## Data Availability

The datasets used and/or analysed during the current study are available from the corresponding author on reasonable request.

## Abbreviations

AAD: Acute aortic dissection
CNKI: China National Knowledge Infrastructure
QUADAS-2: Quality Assessment of Diagnostic Accuracy Studies-2
DOR: Diagnostic odds ratio
+LR: Positive likelihood ratio
−LR: Negative likelihood ratio
SROC: Summary receiver operating characteristics
AUC: Area under the curve
CT: Computerized tomography
MRA: Magnetic resonance angiography
DSA: Digital subtraction angiography
sELAF: Soluble elastin fragments
CRP: C-reactive protein
sm-MHC: Smooth muscle myosin heavy chain
